# Maternal histone acetyltransferase KAT8 is required for porcine preimplantation embryo development

**DOI:** 10.18632/oncotarget.21657

**Published:** 2017-10-06

**Authors:** Zubing Cao, Ronghua Wu, Di Gao, Tengteng Xu, Lei Luo, Yunsheng Li, Jianyong Han, Yunhai Zhang

**Affiliations:** ^1^ Anhui Province Key Laboratory of Local Livestock and Poultry, Genetical Resource Conservation and Breeding, Department of Animal Science, College of Animal Science and Technology, Anhui Agricultural University, Hefei, China; ^2^ State Key Laboratory for Agro-Biotechnology, Department of Biochemistry and Molecular Biology, College of Biological Sciences, China Agricultural University, Beijing, China

**Keywords:** KAT8, H4K16ac, porcine embryos, trophectoderm, genome integrity

## Abstract

K (lysine) acetyltransferase 8 (KAT8), an acetyltransferase that specifically catalyzes histone H4 lysine 16 acetylation, is critical for key biological processes including cell proliferation and maintenance of genome stability. However, the role of KAT8 during preimplantation development in pigs remains unclear. Results herein showed that *KAT8* mRNA is maternally derived and it is required for successful development of early embryos. An abundance of *KAT8* transcripts are expressed in oocytes and its abundance continuously decreases throughout meiotic maturation and preimplantation development. In addition, *KAT8* expression is insensitive to RNA polymerase II inhibitor after embryonic genome activation, suggesting its maternal origin. The levels of *KAT8* mRNA and H4K16 acetylation were effectively knocked down by siRNA microinjection. Knockdown of *KAT8* significantly reduced the blastocyst formation rate and total cell number per blastocyst. Analysis of trophectoderm lineage and marker of DNA double-strand breaks revealed that the impaired developmental competence and quality of embryos might be attributed to defects in both the first two lineages development and genome integrity. Taken together, these results demonstrate that maternal KAT8 is indispensible for porcine early embryo development potentially through maintaining the proliferation of the first two lineages and genome integrity.

## INTRODUCTION

The pig is not only an economically important farm animal in agriculture, but also a considerably better animal model for human disease in biomedicine [1]. The demands of the over-expanding world population for genetically modified pigs with either superior economic traits or human disease state have been increasing. The *in vitro* production (IVP) of pig embryos through assisted reproductive technologies including *in vitro* fertilization (IVF) and somatic cell nuclear transfer (SCNT) is a critical step in the process of creating genetically modified pigs [2]. However, the preimplantation developmental competence of porcine embryos derived from IVP is extremely poor [3]. Therefore, to improve developmental efficiency of porcine embryos, we have to fully understand molecular mechanisms that regulate porcine preimplantation embryo development. It is noteworthy that porcine early embryos not only need to maintain the normal proliferation and differentiation of lineages, but also require a mechanism to protect genomic integrity to guarantee successful preimplantation development. A growing body of evidence strongly shows that maternally derived gene products including transcription factors and epigenetic modifiers tightly regulate these cellular and molecular events in mammalian preimplantation embryos [4]. Indeed, previous studies indicate that some maternal-effect genes are involved in the regulation of preimplantation embryo development in mice [5–7] and pigs [8, 9]. For example, maternally provided BCAS2 knockout embryos exhibited severe developmental defects mainly because of the accumulation of DNA double-strand breaks [10].

In addition, histone acetylation is also an important epigenetic modification involved in controlling cell cycle progression and maintenance of genomic stability in somatic cells [11, 12]. It is controlled by the opposing activity of histone acetyltransferases (HATs) and histone deacetyltransferases (HDACs) [13]. Specifically, the acetylation of histone H4 lysine 16(hereafter referred to as H4K16ac) is correlated with transcription activation, cell cycle progression and DNA damage repair [14–16]. It has been implied that H4K16ac levels must maintain the dynamic and precise equilibrium in mouse oocytes. H4K16 hyperacetylation induced by *Hdac2* null mutation led to defects in spindle assembly and chromosome segregation of oocytes in mice [17], while complete loss of H4K16 acetylation triggered by *Esco2* depletion also caused defects in mouse oocyte meiotic maturation [18].

KAT8 (also called as MOF or MYST1) is an acetyltransferase specific for catalyzing the acetylation of histone H4 lysine 16 residues in mammalian cells [14]. It is reported that KAT8 is expressed in many types of tissues and implicated in several biological processes including DNA replication, DNA repair, cell cycle and tumorgenesis [19]. Moreover, it has been discovered that KAT8 not only maintains self-renewal and pluripotency of mouse embryonic stem cells [20], but also facilitates the generation of induced pluripotent stem cells in human [21], suggesting KAT8 may be related to the establishment of both totipotency and pluripotent lineage in embryos. However, zygotic *KAT8* knockout embryos did not display preimplantation developmental phenotype in mice [22, 23]. It is speculated that the functional role of KAT8 during mouse preimplantion embryo development could be covered by the preexisting maternal KAT8 during oocyte growth. Because zygotic *KAT8* depleted mouse embryos still could develop into blastocysts with high levels of H4K16ac [22], but RNAi-mediated depletion of maternal *KAT8* could accelerate loss of both KAT8 and H4K16ac in mouse 4-cell embryos [24], indicating that maternal KAT8 indeed exists in mouse preimplantation embryos. Correspondingly, the newest study showed that *KAT8* is highly expressed in mouse oocytes and maternal *KAT8* knockout caused developmental arrest and death of oocytes and follicles due to apoptosis [25]. Although maternal KAT8 is involved in the regulation of oocyte and follicle development in mice, whether maternally inherited KAT8 exists and its role in porcine preimplantation embryos remains unclear.

Here, we characterized the expression pattern of *KAT8* during porcine oocyte maturation and early embryo development and demonstrated that *KAT8* mRNA in porcine early embryos is of maternal origin. Subsequently, we used RNAi to specifically deplete maternal *KAT8* in porcine early embryos. We found that the majority of *KAT8*-depleted embryos arrested at the morula stage probably due to severe defects in lineage proliferation and genomic integrity. Our findings demonstrate that maternally inherited KAT8 is essential for porcine preimplantion embryo development.

## RESULTS

### Cloning and characterization of porcine *KAT8*

**Figure 1:**
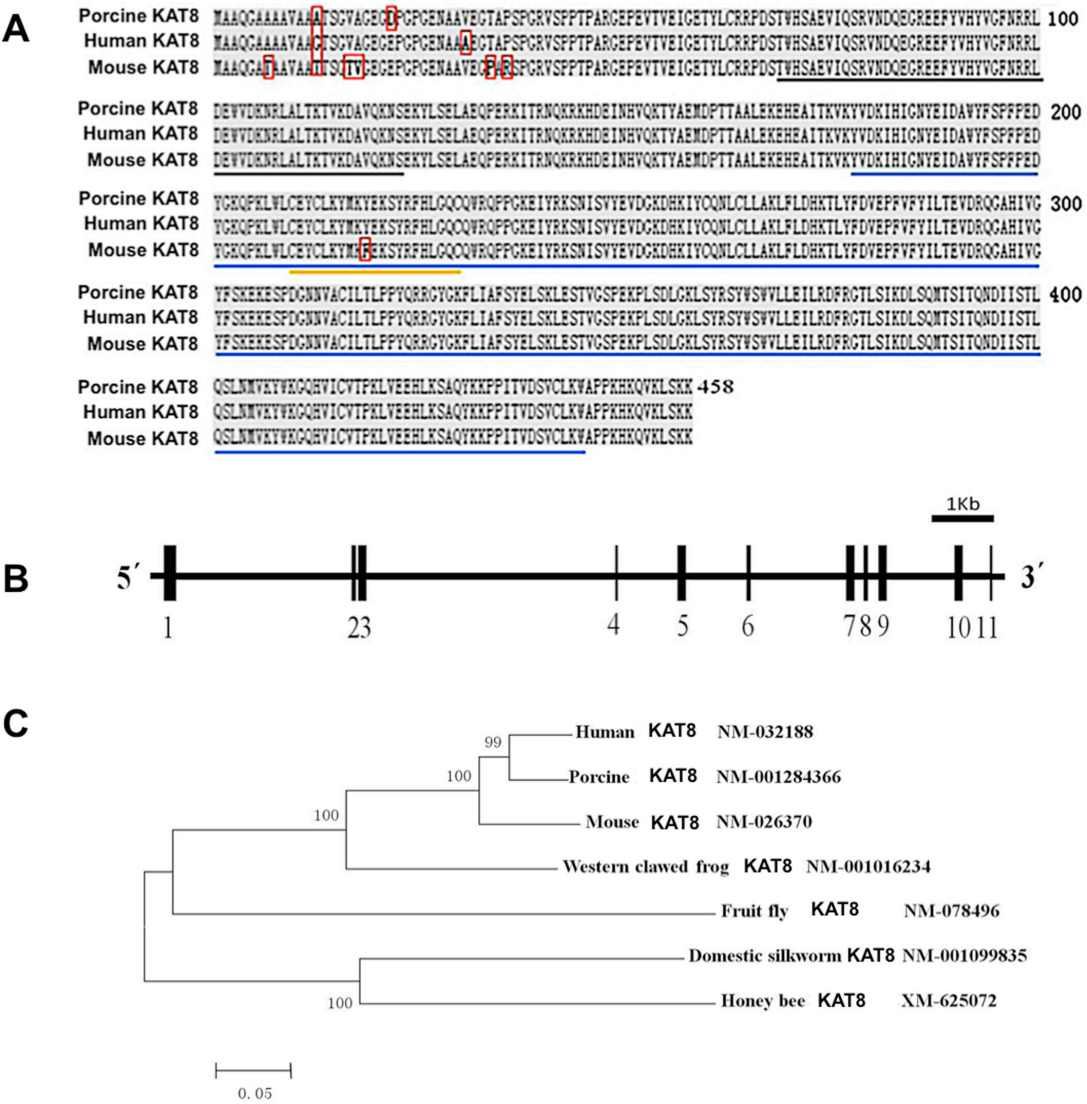
Genomic organization and homologous analysis of porcine *KAT8* **(A)** The amino acid sequences of KAT8 from porcine, human and mouse were aligned by DNAMAN software. Identical amino acids are shaded in black. The sequences in red box are different between three species. The conserved domains are underlined in black, blue and yellow, respectively. **(B)** Schematic diagram of genomic structure of porcine *KAT8*. Solid boxes and horizontal lines represent the exons and introns, respectively. The transcript corresponding to p*KAT8* consists of 11 exons separated by 10 introns. **(C)** Phylogenetic tree of *KAT8* genes based on available KAT8 amino acid sequence from various organisms is drawn using MEGA 5.2 software.

Because porcine *KAT8* (hereafter referred to as p*KAT8*) has not been annotated in the NCBI database, we cloned full-length cDNA sequence encoding p*KAT8* by RT-PCR using primers designed based on the predicted sequences. The size of p*KAT8* cDNA was confirmed by agarose gel electrophoresis (Supplementary Figure 1). The complete sequences of p*KAT8* cDNA have been deposited in the GenBank database with the accession number (NM_001284366). The cDNA is 1,471 bp in length, which contains an open reading frame of 1,374 bp encoding a protein of 458 amino acids with a conserved acetyltransferase domain (Figure 1A). The pKAT8 protein shares 99% and 98% sequence identity with those of human and mouse as shown in the Supplementary Table 1, respectively, which indicates KAT8 is highly conserved across these species. Additionally, a genomic database query revealed that the p*KAT8* gene contains 11 exons separated by 10 introns and is located at chromosome 3, spanning over 10.9 kb in length (Figure 1B). In addition, phylogenetic analysis of multiple species showed that pKAT8 has a closer relationship with human than with other species (Figure 1C).

### *KAT8* expression in porcine gametes, different tissues and early embryos

**Figure 2:**
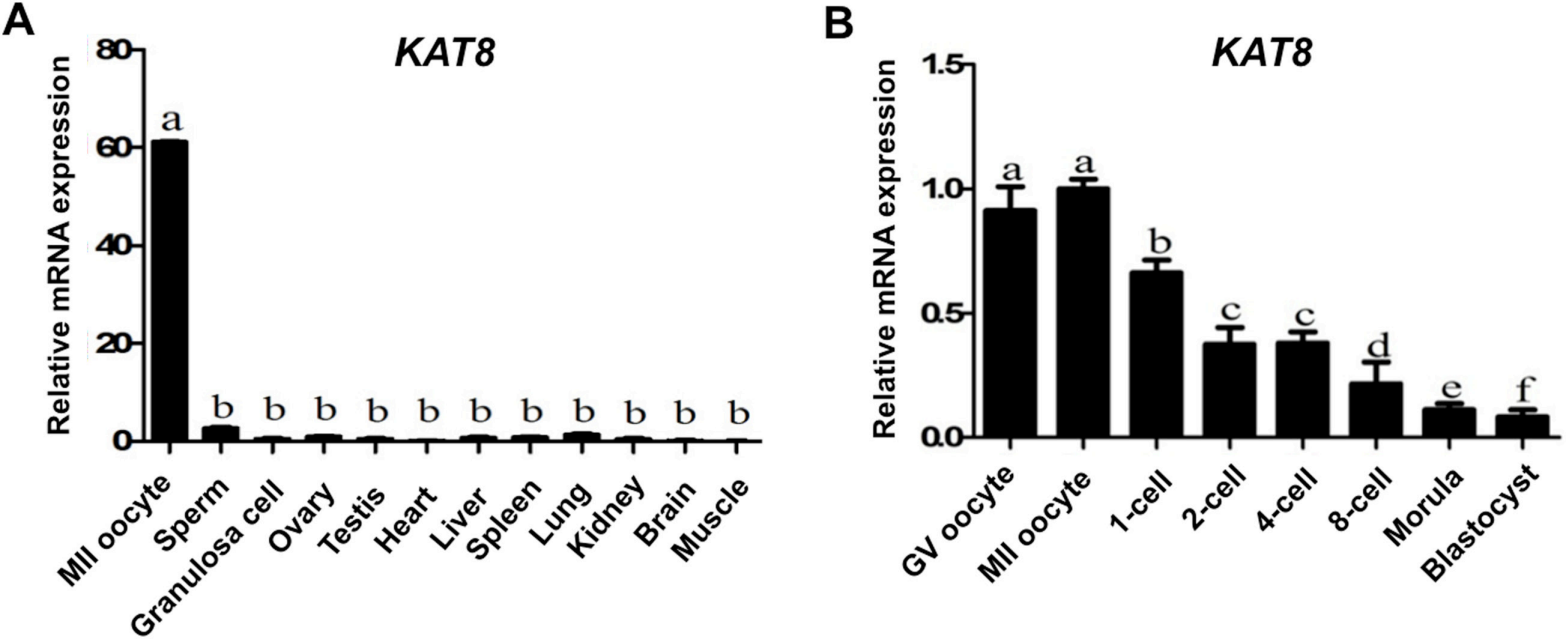
Relative abundance of *KAT8* in porcine gametes, different tissues and early embryos **(A)** *KAT8* expression in oocytes, sperm, granulosa cells and different tissues. Relative levels of *KAT8* mRNA were determined by qPCR from three independent replicates. Data were normalized against endogenous housekeeping gene *EF1α1* and the value from ovary was set as one. Values are shown as mean ± S.E.M and different letters indicate significant differences (*P* < 0.05). **(B)** *KAT8* expression in early embryos. Relative levels of *KAT8* transcripts were determined by qPCR from three independent replicates. Data were normalized against endogenous housekeeping gene *EF1α1* and the value from MII oocyte was set as one. Values are shown as mean ± S.E.M and different letters across stages indicate significant differences (*P* < 0.05).

To determine the relative expression of *KAT8* in MII oocytes, sperm, granulosa cells, testis, ovary and several non-reproductive tissues such as heart, liver, spleen, lung, kidney, brain and muscle, *KAT8* transcripts were analyzed by qPCR. Results revealed that *KAT8* mRNA was abundantly expressed in MII oocytes, but slightly detectable in sperm, granulosa cells and other tissues. Also, the levels of *KAT8* transcripts in MII oocytes were significantly higher than that in other cells and tissues (*P* < 0.05) (Figure 2A). These data suggest that *KAT8* is likely a maternal gene as it is abundantly expressed in MII oocytes compared with granulosa cells. To further characterize the dynamic expression of *KAT8* mRNA during porcine oocyte maturation and early embryonic development, we performed qPCR to analyze *KAT8* transcripts in GV, MII oocytes, 1-cell, 2-cell, 4-cell, 8-cell, morula and blastocysts. qPCR results showed that the abundance of *KAT8* transcripts was high in GV and MII oocytes (*P* < 0.05) (Figure 2B). After parthenogenetic activation, the levels of *KAT8* mRNA gradually declined from pronuclear stage onwards and eventually reached to a minimum at the blastocyst stage (*P* < 0.05) (Figure 2B). Consistent with this finding, mouse *KAT8* also exhibits similar expression pattern to pig at the same developmental period (Supplementary Figure 2) [26]. In addition, the expression pattern of *KAT8* mRNA during meiotic maturation and early embryonic development is similar to other known maternal-effect genes essential for early embryogenesis [5, 6, 8, 15, 27, 28], suggesting its potential maternal origin of *KAT8* transcripts.

### Maternal inheritance of *KAT8* transcripts during porcine early embryo development

**Figure 3:**
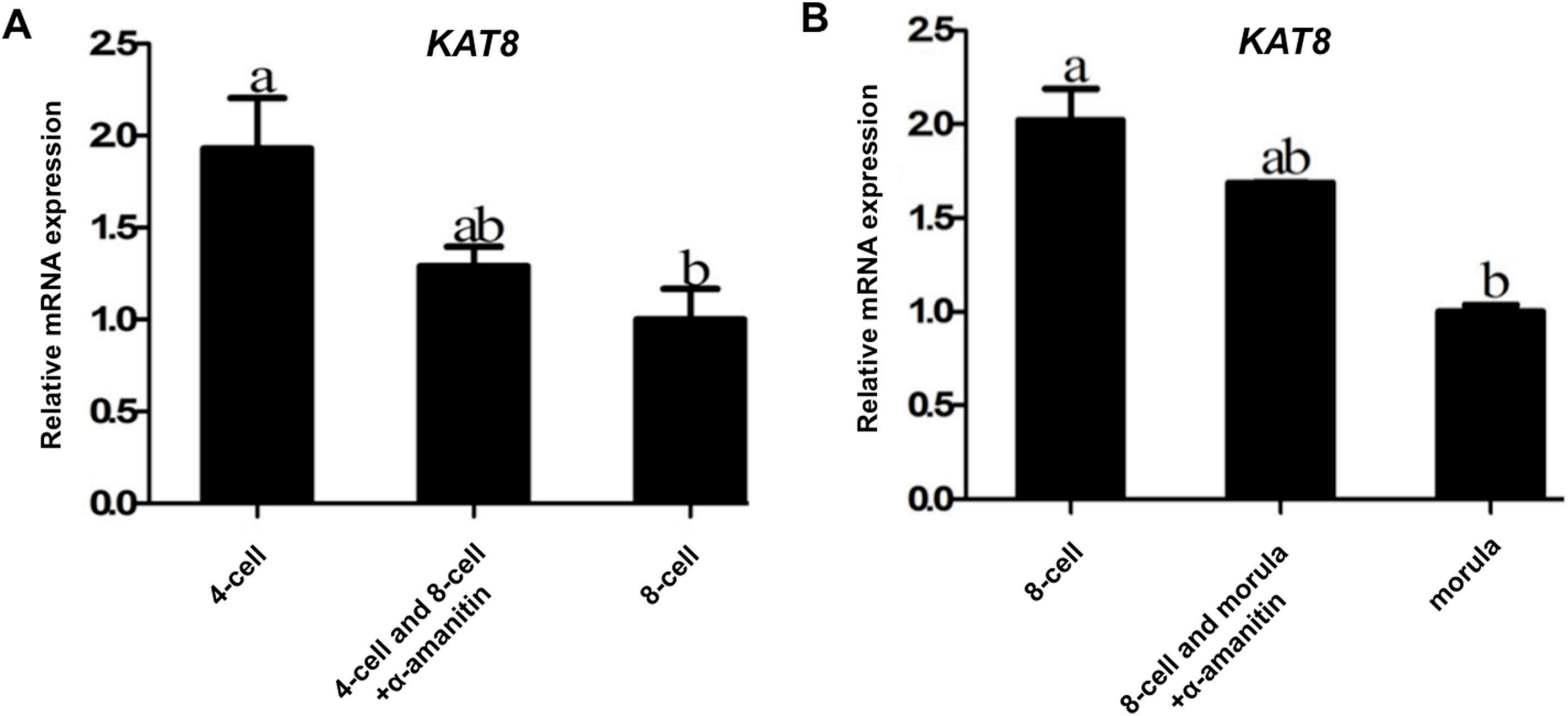
Confirmation of maternal origin of *KAT8* expression **(A)** *KAT8* expression in control 4-cell, 8-cell and α-amanitin-treated 4-cell and 8-cell embryos. Relative levels of *KAT8* mRNA were determined by qPCR from three independent replicates. Data were normalized against endogenous housekeeping gene *EF1α1* and the value from control 8-cell embryos was set as one. Values are shown as mean ± S.E.M and different letters across groups indicate significant differences (*P* < 0.05). **(B)** *KAT8* expression in control 8-cell, morula and α-amanitin-treated 8-cell and morula. Relative levels of *KAT8* transcripts were determined by qPCR from three independent replicates. Data were normalized against endogenous housekeeping gene *EF1α1* and the value from control morula was set as one. Values are shown as mean ± S.E.M and different letters indicate significant differences (*P* < 0.05).

To further address whether *KAT8* mRNA in early embryos is fully derived from oocytes, 4-cell and 8-cell embryos were cultured for 24 h in PZM-3 medium containing 25 μg/ml α-amanitin (an inhibitor of RNA polymerase II) to discriminate between maternal and zygotic *KAT8* transcripts. qPCR results revealed that the levels of *KAT8* mRNA in control 4-cell embryos were significantly lower than that in control 8-cell embryos (*P* < 0.05). Importantly, the relative abundance of *KAT8* mRNA did not change in 8-cell embryos comparing control to α-amanitin treated group (Figure 3A). Likewise, *KAT8* showed a significant reduction in the mRNA level during the transition from 8-cell to morula stage (*P* < 0.05), but α-amanitin treatment did not further decrease the expression levels of *KAT8* at the same time points (Figure 3B). These results indicate that the expression of *KAT8* in early embryos is insensitive to the inhibition of RNA polymerase II. Therefore, these data strongly demonstrate that *KAT8* transcripts are maternally inherited products during porcine early embryo development.

### RNAi-mediated efficient knockdown of *KAT8* and its catalyzed product H4K16ac in early embryos

**Figure 4:**
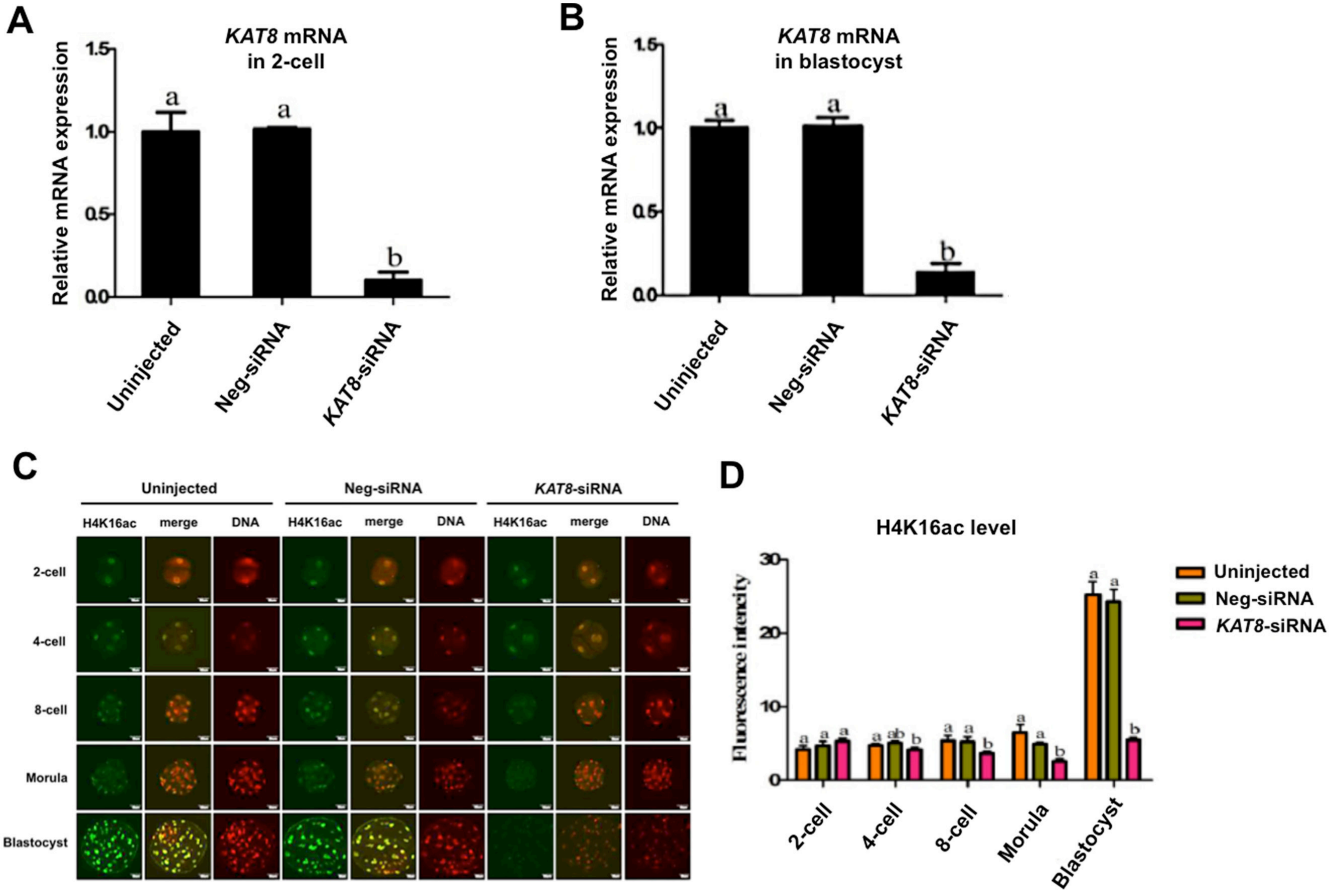
Validation of the efficiency of RNAi-mediated *KAT8* knockdown in porcine embryos *KAT8* expression was analyzed by qPCR in non-injected, neg-siRNA and *KAT8* siRNA-injected 2-cell embryos **(A)** and blastocysts **(B)**. All experiments were independently repeated three times. Data were normalized against endogenous housekeeping gene *EF1α1* and the value from non-injected embryos was set as one. Values are shown as mean ± S.E.M and different letters across groups indicate significant differences (*P* < 0.05). **(C)** Immunofluorescence staining of embryos at different developmental stages. Embryos in non-injected, Neg-siRNA and *KAT8* siRNA-injected groups were stained for H4K16ac (green) and DNA (propidium iodide, red). Representative fluorescence images are shown. Middle panel in each group shows the merged images (yellow) between H4K16ac signal and DNA staining. Scale bar: 50 μm. **(D)** Quantification of H4K16ac fluorescence intensity in embryos of different developmental stages. Yellow bar demotes non-injected group, green bar marks Neg-siRNA injected group, pink bar represents *KAT8* siRNA-injected group. Data are shown as mean ± S.E.M and different letters across groups indicate significant differences (*P* < 0.05).

To elucidate the functional role of *KAT8* during porcine preimplantation embryo development, we used an RNAi approach to deplete maternally derived *KAT8* transcripts. MII oocytes were microinjected with either 50 μM *KAT8* or negative control siRNA (hereafter referred to as Neg-siRNA); non-injected oocytes were used as an additional control. The oocytes from three groups were then parthenogenetically activated (PA) and cultured for 2 to 7 days. qPCR results revealed that microinjection of *KAT8* siRNA resulted in a more than 90% reduction of *KAT8* mRNA levels at 2-cell and blastocyst stages, respectively (Figure 4A and 4B). Unfortunately, because of the lack of porcine specific KAT8 antibodies, we could not directly assess the effect of *KAT8* siRNA injection on KAT8 protein levels. However, it has been recently reported that H4K16 is a sole catalyzed target of KAT8 protein in mouse oocytes [25]. As an alternative to direct evaluation of KAT8 protein levels, we thus examined the H4K16ac levels to validate the knockdown efficiency of *KAT8* siRNA. The specificity of the commercially available H4K16ac antibody was verified by a pre-absorption test using the antigen peptide in PA blastocysts (Supplementary Figure 3). Immunofluorescence staining was performed to examine the H4K16ac levels in porcine embryos at different developmental stages (Figure 4C). The quantitative result of fluorescence intensity showed that H4K16ac levels were significantly reduced in *KAT8* siRNA-injected embryos from 4-cell stage onwards compared to controls (Figure 4D) (*P* < 0.05). Altogether, these results suggest that *KAT8* siRNA can carry out the robust reduction of levels of *KAT8* and its catalyzed product H4K16ac in early embryos.

### *KAT8* knockdown reduces the developmental competence and quality of early embryos

**Figure 5:**
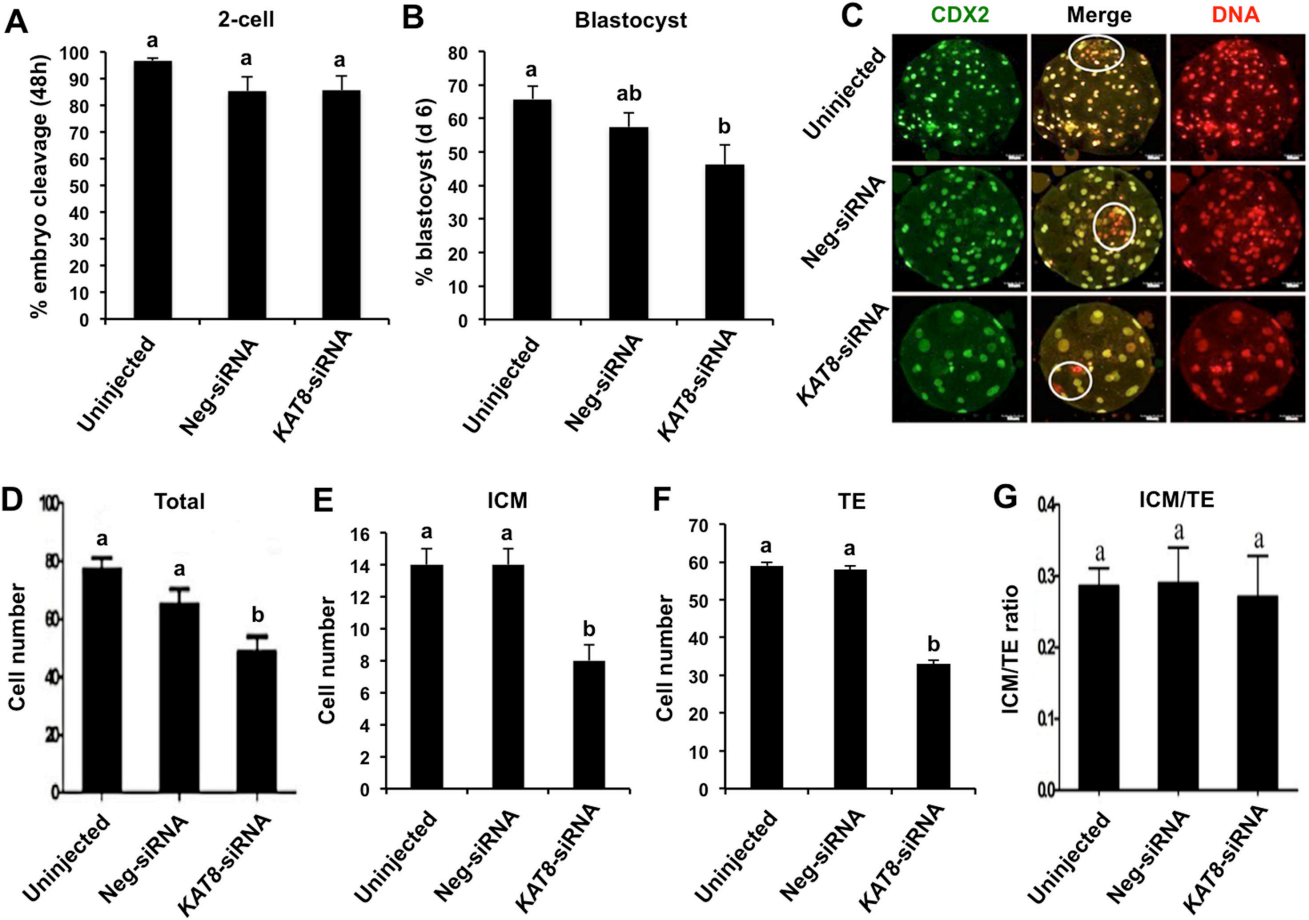
Effect of *KAT8* knockdown on porcine early embryo development and quality MII oocytes were microinjected with *KAT8* siRNA. Non-injection and neg-siRNA injection were set as two control groups. MII oocytes from each group (n=228, 220 and 230) were parthenogenetically activated and cultured up to the blastocyst stage. The number of 2-cell (48 h post PA, n=220, 188 and 197) **(A)**, blastocysts (day 7 post PA, n=150, 126 and 106) **(B)** were recorded and statistically analyzed. All experiments were independently repeated six times. Data are shown as mean ± S.E.M and different letters across groups indicate significant differences (*P* < 0.05). **(C)** Immunofluorescence staining of blastocysts in each group with CDX2 antibody. Representative fluorescence images are shown with CDX2 (green) and DNA (red). Middle panel shows the merged images between CDX2 signal and DNA staining. White circle denotes ICM region. Scale bar: 50μm. **(D, E, F** and **G)** Effect of *KAT8* knockdown on cell number and lineage allocation of the resulting blastocysts. The average number of total cells (n=75, 63 and 53) (D), ICM cells (E), TE cells (F) and ratio of ICM: TE cell number (G) was statistically analyzed, respectively. Data are shown as mean ± S.E.M and different letters across groups indicate significant differences (*P* < 0.05). ICM: inner cell mass, TE: trophectoderm.

To evaluate whether *KAT8* knockdown has an effect on porcine preimplantation embryo development, blastomere cleavage and blastocyst formation were recorded. We found that *KAT8* knockdown did not affect the cleavage rate (Figure 5A), but the rate of blastocyst formation was significantly reduced compared to uninjected group (Figure 5B) (*P* < 0.05). To further ascertain the quality of embryos that developed to blastocyst stage, we determined the number of trophectoderm (TE) lineage-specific and total cells in the resulting blastocysts from each group by immunostaining against CDX2 as marker of TE cells and propidium iodide to counterstain DNA in all cells (Figure 5C). The number of inner cell mass (ICM) cells was indirectly determined by subtracting the CDX2-positive cell number from total cell number. Results showed that the total cell number of blastocysts produced from *KAT8* siRNA-injected oocytes was significantly reduced compared to that in uninjected control and neg-siRNA group (Figure 5D) (*P* < 0.05), and these blastocysts also had fewer ICM (Figure 5E) and TE cells (Figure 5F) than two control groups (*P* < 0.05). On the other hand, the ratio of ICM: TE cells was similar among three groups (Figure 5G). Therefore, these results demonstrate that an impaired ability of porcine oocytes to develop into blastocysts after depleting *KAT8*, indicating an important functional requirement of *KAT8* during porcine preimplantation embryo development.

### *KAT8* knockdown causes DNA damage in porcine blastocysts

**Figure 6:**
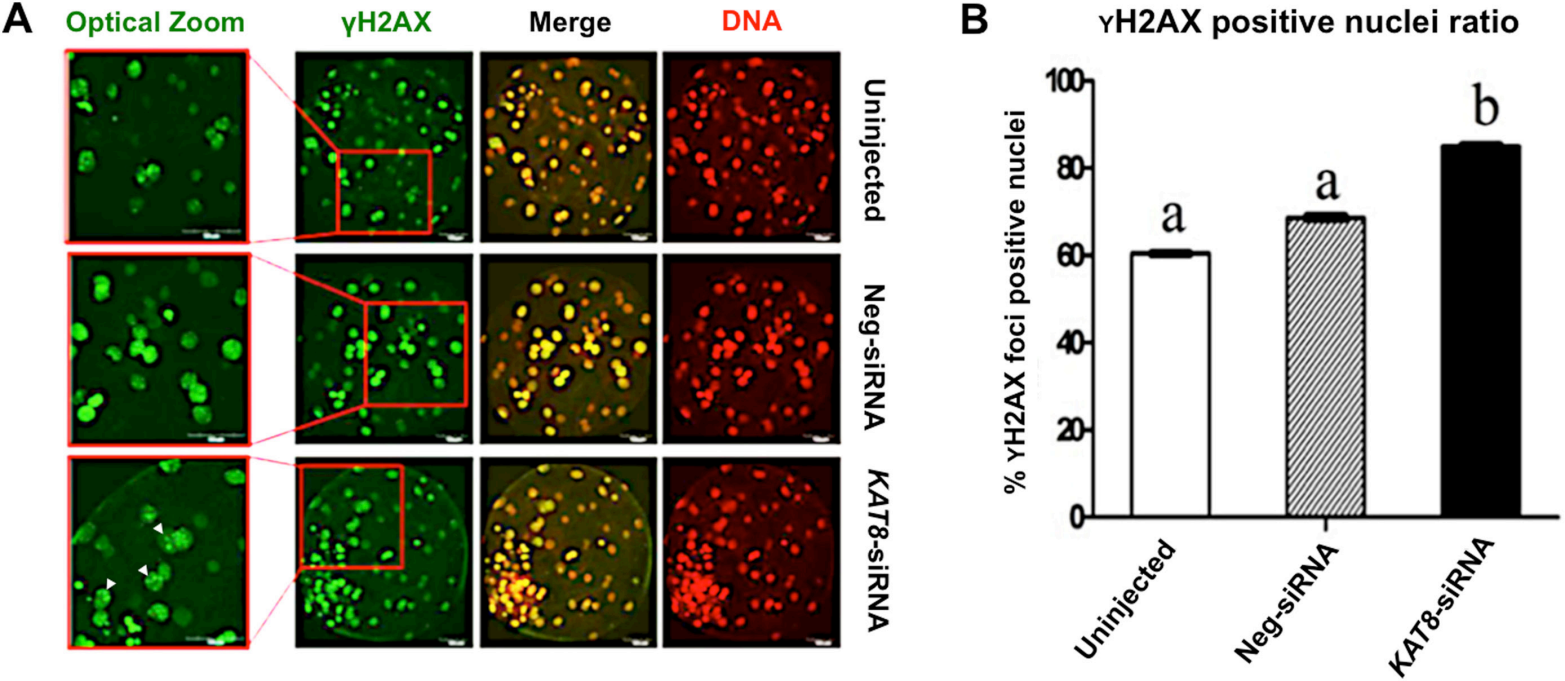
*KAT8* knockdown induces DNA damage in porcine blastocysts **(A)** Immunofluorescence staining of γH2AX. Blastocysts in uninjected, Neg-siRNA and *KAT8* siRNA-injected groups were stained for γH2AX (green) and DNA (propidium iodide, red). The experiment was independently repeated three times. Representative fluorescence images are shown. Middle panel in each group shows the merged images (yellow) between γH2AX signal and DNA staining. Red solid insets indicate cells shown at higher magnification on the left. White arrowheads mark γH2AX foci positive cells. Scale bar: 50 μm. **(B)** Analysis of the ratio of γH2AX foci positive cells in blastocysts from three groups. The number of γH2AX foci positive and total cells of blastocysts (n=75, 63 and 53) was counted separately and the ratio of γH2AX foci positive:total cells was statistically analyzed. Data are shown as mean ± S.E.M and different letters across groups indicate significant differences (*P* < 0.05).

Given that the acetylation of histone H4 lysine 16 residues is critical for DNA damage response (DDR) and DNA double-strand breaks (DSBs) repair [15], we thus hypothesized that loss of H4K16ac could lead to a defective DDR and an accumulation of unrepaired DSBs in porcine early embryos. To address this, we used the resulting blastocysts produced from *KAT8* siRNA-injected oocytes to perform the immunofluorescence staining of γH2AX, a marker of DNA double-strand breaks (Figure 6A). The quantitative analysis of γH2AX foci indicated that *KAT8* knockdown indeed resulted in the increased number of γH2AX positive foci nuclei relative to uninjected and neg-siRNA injected groups (Figure 6B), suggesting genomic destruction of embryos after depletion of *KAT8*. Hence, these results imply that KAT8 is involved in the maintenance of genomic integrity during porcine early embryo development.

## DISCUSSION

In the present study our results reveal an important role of maternally provided histone acetyltransferase KAT8 in porcine preimplantation embryos. Knockdown of *KAT8* by microinjecting siRNA to MII oocytes resulted in the lower developmental competence of oocytes developed into blastocysts. The reduced rate of blastocyst formation may be due to defects in the first two lineages (ICM and TE) proliferation and genomic integrity. Therefore, the results here indicate that maternal KAT8 functions during porcine early embryo development.

KAT8 is a histone acetytransferase that specifically acetylates histone H4 lysine 16 (H4K16) in mammalian cells and plays a role in several cellular biological processes including DNA replication, DNA repair and cell cycle [24]. However, cDNA sequences of porcine *KAT8* have been not cloned before now, in this study we successfully cloned the full-length sequences of porcine *KAT8* cDNA. Cloning and acid amino sequence alignment of porcine *KAT8* indicates that it shares three similar domain structures and is also highly conserved compared to other species, which imply that KAT8 may have a similar function between different species. The relative expression of *KAT8* in porcine different tissues revealed that *KAT8* is highly expressed in MII oocytes compared to sperm and other tissues, suggesting that the expression of *KAT8* is oocyte-specific. To our knowledge, the outcome is the first report of histone acetytranseferase *KAT8* gene present restrictedly in porcine oocytes. The pattern of *KAT8* expression during porcine preimplantation embryo development is consistent with that of a maternal effect gene, with high levels in GV and MII oocytes that gradually decrease from pronuclear to blastocyst stage. Microarray analysis indicated that the expression profile of *KAT8* in mouse oocytes and preimplantation embryos is similar to that in pigs [26]. Furthermore, the levels of *KAT8* mRNA observed at 4-cell and 8-cell stage were not significantly diminished by transcriptional inhibition, demonstrating the maternal origin of *KAT8* transcripts in porcine early embryos. In contrast, α-amanitin treatment resulted in slightly increased *KAT8* transcript abundance, suggesting that degradation or recruitment of *KAT8* mRNA in porcine early embryos may be transcription-dependent.

Previous studies indicated that maternal factors including mRNA, non-coding RNA and proteins play an important role in preimplantation embryo development [4]. Indeed, our and others’ studies recently demonstrated that several maternal genes, namely *DJ-1*, *VIMENTIN*, *SEBOX* and *WDR5*, are essential for successful preimplantation development of porcine cloned and *in vitro* fertilized embryos [8, 9, 29, 30]. To further characterize the function of *KAT8* as a maternal gene in porcine early embryos, we knocked down *KAT8* via microinjection of siRNA into MII oocytes and then evaluated developmental efficiency of embryos. Results showed that *KAT8* knockdown did not decrease the cleavage rate, but significantly reduced the blastocyst rate. It should be noted that a part of *KAT8*-siRNA oocytes developed into blastocysts could be due to siRNA degradation or insufficient injection amount of *KAT8* siRNA.

A closer analysis of the quality of the resulting blastocysts produced from *KAT8*-siRNA injected oocytes revealed that total cell number per blastocyst and cell number of the first two lineages including ICM and TE were apparently decreased, but the ratio of ICM: TE did not change. It has been reported that the acetylation of histone H4 at K16 by KAT8 is an essential epigenetic signature of cellular proliferation in mouse embryogenesis [22]. Moreover, in mouse preimplantation embryos, histone variant H3.3 depletion resulted in the significant reduction in H4K16ac levels and highly condenses of linker histone H1, which indirectly induced the arrest of cell cycle progression [24]. Thus, *KAT8* knockdown-induced reduction of H4K16ac levels may cause cell cycle arrest that could likely account for the lower number of total cells and ICM and TE lineage cells in these impaired blastocysts. On the other hand, knockout of *KAT8* led to differentiation and pluripotent state loss of mouse embryonic stem cells [20] and *KAT8* depletion also resulted in loss of epithelial cell features [31], which may provide alternative explanation for reduction in the numbers of ICM and TE lineage cells. Altogether, the proliferation defects in the two types of lineage cells could significantly impair porcine blastocyst formation.

Two previous studies showed that KAT8 in mouse oocytes and blastocysts specifically acetylates H4K16, but not other lysine residues of histone H3 and H4 [23, 25]. Based on these studies, we examined the levels of H4K16ac in *KAT8* depleted embryos; results revealed that H4K16ac levels were significantly reduced in *KAT8* knockdown embryos. Given reduction of H4K16ac levels is correlated with DDR and DSBs repair [15], we thus hypothesized that *KAT8* depleted embryos might accumulate many DSBs. Histone H2AX is phosphorylated at the site of DNA damage and then phosphorylated form of H2AX (also called as γH2AX) is usually used as a marker of DNA double-strand breaks [32]. As expected, there was an increased incidence of γH2AX positive foci nuclei observed in *KAT8* knockdown blastocysts, suggesting that *KAT8* depletion indeed resulted in unrepaired DNA damage in porcine blastocysts. Consistent with our work, *KAT8* knockout oocytes and embryos also exhibited a large number of γH2AX in mice [23, 25]. Importantly, a recent study showed that increased levels of γH2AX dramatically reduced developmental efficiency of porcine preimplantion embryos [33], which may partly explain for the poor early development of *KAT8* knockdown porcine embryos observed in the present study.

In conclusion, maternally inherited KAT8 is indispensible for porcine preimplantation embryo development probably through maintaining the first two lineages proliferation and genome integrity.

## MATERIALS AND METHODS

All chemicals in this study were purchased from Sigma (Sigma-Aldrich, St Louis, MO) unless otherwise stated. All experiments were conducted in accordance with the Institutional Animal Care and Use Committee (IACUC) guidelines under current approved protocols at Anhui Agricultural University. This study had been fully reviewed and approved by IACUC.

### Cloning of porcine *KAT8*

PCR primers were designed based on the predicted cDNA sequence for the pig KAT8 (p*KAT8*) gene in the National Center for Biotechnology Information Database (NCBI). Primer information is shown in Supplementary Table 2. The full-length coding sequence of porcine *KAT8* was amplified from porcine reference cDNA synthesized from blastocysts. The amplified PCR products were cloned using a TOPO-TA cloning kit (Invitrogen) and completely sequenced.

### Oocyte *in vitro* maturation (IVM)

The experiment was conducted as described previously [34]. Briefly, ovaries from sows were collected at a local slaughterhouse and transported to the laboratory at 28°C-35°C. The follicular fluid from follicles with 3 to 6 mm in diameter was aspirated using a sterile 10 mL syringe. The cumulus-oocyte complexes (COCs) with more than three layers of cumulus cells and homogeneous ooplasm were selected under a stereomicroscope. Subsequently, the COCs were cultured in IVM medium (TCM-199 supplemented with 15% FBS, 10 ng/mL EGF, 10% porcine follicular fluid, 10 IU/mL eCG, 5 IU/mL hCG, 0.8 mM L-glutamine and 0.05 mg/mL gentamicin) for 42-44 h at 38.5°C, 5% CO_2_ and saturated humidity. Cumulus cells enclosed outside the COCs were removed using1 mg/mL hyaluronidase solution. Finally, oocytes containing the first polar body (pb1) were selected for subsequent experiments.

### Parthenogenetic activation

Oocytes with the first polar body were washed with activation medium (280 mM mannitol supplemented with 0.1 mM CaCl_2_, 0.1 mM MgCl_2_ and 0.01% polyvinyl alcohol) and then treated with two pulses of direct current (1.56 kV/cm for 80 μs) by using cell fusion instrument (CF-150B, BLS, Hungary). Subsequently, embryos were washed in porcine zygote medium (PZM-3) [35] three times, followed by 4 h of incubation in the chemically assisted activation medium (PZM-3 supplemented with 10 μg/mL cycloheximide and 10 μg/mL cytochalasin B). Embryos were then washed three times with PZM-3 medium and cultured in fresh PZM-3 medium at 38.5°C, 5% CO_2_ and 95% air with saturated humidity.

### Microinjection

To deplete endogenous *KAT8* mRNA, small interfering RNA (siRNA) of *KAT8* was microinjected into the cytoplasm of porcine MII stage oocytes. Two *KAT8* siRNAs were purchased to target different coding regions of porcine *KAT8* and a non-specific siRNA was used as a negative control (GenePharma, China). All siRNA sequence information used in this study is shown in Supplementary Table 3. Microinjection was performed in T2 (TCM199 with 2% FBS) medium with 7.5 μg/ml cytochalasin B on the heated stage of an inverted microscope (Olympus, Japan). Approximately 10 pl siRNA solution was microinjected into cytoplasm of MII stage oocytes, manipulated oocytes were parthenogenetically activated after recovering for 30 min in PZM-3 and then cultured *in vitro*. Two control groups (no injection, non-specific siRNA injection) were designed to test potential effects of the microinjection technique and siRNA toxicity on embryonic development.

### Immunofluorescence staining

The experiment was performed as described previously [36]. Briefly, zona pellucida of embryos were removed using 0.5% pronase solution. Embryos were fixed in 4% paraformaldehyde solution for 15 min and permeabilized in 0.5% Triton X-100 solution for 30 min at room temperature, then washed briefly with DPBS containing 0.3% PVP. Embryos were blocked in 2% BSA solution overnight at 4°C and subsequently incubated for 1 h in blocking buffer containing primary antibodies against H4K16ac (Abcam, ab61240, 1:200), CDX2 (BioGenex, AM392, 1:20) and γH2AX (Abcam, ab26350, 1:200) at room temperature. After washing several times, embryos were incubated for 1 h in blocking solution containing secondary antibodies including goat anti-Rabbit IgG conjugated with Alexa Fluor 488 (Molecular probe, A11008, 1:200) or goat anti-mouse IgG conjugated with Alexa Fluor 488 (Molecular probe, A11029, 1:200) in the dark at room temperature. Finally, embryos were washed several times and mounted on glass slides with a small drop of Vectashield (VectorLab) mounting medium, then covered by a glass coverslip and imaged using confocal laser scanning microscopy (Olympus, FluoView1000). Embryos omitting primary antibody were used as negative control to examine the specificity of the reaction.

### Quantitative analysis of fluorescence intensity

The signal intensity for H4K16ac in embryos was analyzed as described previously [36]. Briefly, nuclei of blastomeres were localized by DAPI staining using the 40 × objective lens. Using the same setting parameters including magnification fold, exposure time, brightness and contrast, fluorescence images were captured by Olympus FluoView 1000 software. Quantitative assessment of signal intensity in nuclear or cytoplasmic areas was performed using Image J software (1.49v, NIH). The border around the nuclei was manually delineated according to DNA staining. Moreover, at least three different cytoplasmic areas were selected for normalization to background. The average pixel intensity of the nuclear areas was calculated by Image J, and then normalized by dividing by the average pixel intensity of the cytoplasmic areas.

### RNA extraction and reverse transcription

For each biological replicate, total RNA was isolated from the pooled oocytes, embryos (n=10) or other tissues using RNeasy Micro Kit (Qiagen, 74004). The extracted RNA was quantified with spectrophotometry at 260/280 nm with a NanoDrop 2000 instrument (Thermo Scientific, Waltham, MA, USA). Reverse transcription was immediately performed using QuantiTect Reverse Transcription Kit (Qiagen, 205311). The cDNA was aliquoted and stored at -80°C until ready for use. The samples were collected three times and three biological replicates were conducted.

### Quantitative real-time PCR

The primers used in the present study are shown in Supplementary Table 2. Polymerase chain reaction was prepared using FastStart SYBR Green Master (Roche, 04673514001) and performed using StepOne Plus (Applied Biosystems). Each reaction consisted of 1.5 μl cDNA, 7.5 μl 2 × SYBR Green PCR master mix, 0.9 μl of each primer pair, and 5.1 μl ultrapure water. The housekeeping gene *EF1α1* was used as an endogenous reference. The following amplification conditions were used: pre-denaturation at 95°C for 10 min, followed by 45 amplification cycles of 95°C denaturation for 15 seconds, 60°C annealing for 10 seconds, and 72°C extension for 20 seconds. Three technical replicates were conducted in each PCR reaction.

### Statistical analysis

All experiments were repeated at least three times. Prior to statistical analysis, the percentage data were subject to arc-sine transformation. SPSS (version 17.0) software was used to do one-way ANOVA of the cleavage rate, blastocyst rate, total cell number per blastocyst, gene expression, fluorescence intensity of H4K16ac and ratio of γH2AX positive nuclei. All data were presented as mean ± standard error of mean (mean ± S.E.M) and *P*<0.05 was considered to be statistically significant.

## SUPPLEMENTARY MATERIALS FIGURES AND TABLES



## Abbreviations

COCs: cumulus-oocyte complexes
KAT8: K (lysine) acetyltransferase 8
GV: germinal vesicle
MII: metaphase II
IVM: *in vitro* maturation
PA: parthenogenetic activation
IVF: *in vitro* fertilization
SCNT: somatic cell nuclear transfer
PZM-3: porcine zygote medium-3
DSBs: DNA double-strand breaks
H4K16ac: histone H4 lysine 16 acetylation
pb1: the first polar body
ICM: inner cell mass
TE: trophectoderm
HATs: histone acetyltransferases
HDACs: histone deacetyltransferases
bp: base pair
kb: kilobase pair
qPCR: quantitative real-time polymerase chain reaction
TCM-199: tissue culture medium-199
FBS: fetal bovine serum
EGF: epidermal growth factor
cDNA: complementary DNA
Neg-siRNA: negative control siRNA
PVP: polyvinyl pyrrolidone
DAPI: 4′, 6-diamidino-2-phenylindole
DPBS: dulbecco's phosphate-buffered saline
