# Supplementary material for: Maternal histone acetyltransferase KAT8 is required for porcine preimplantation embryo development
Source: Oncotarget. 2017 Oct 6;8(52):90250–61. doi: 10.18632/oncotarget.21657 (PMC5685746; doi:10.18632/oncotarget.21657)
Supplement: Supplementary file 1 [file oncotarget-08-90250-s001.pdf]

Maternal histone acetyltransferase KAT8 is required for porcine preimplantation embryo development

SUPPLEMENTARY MATERIALS

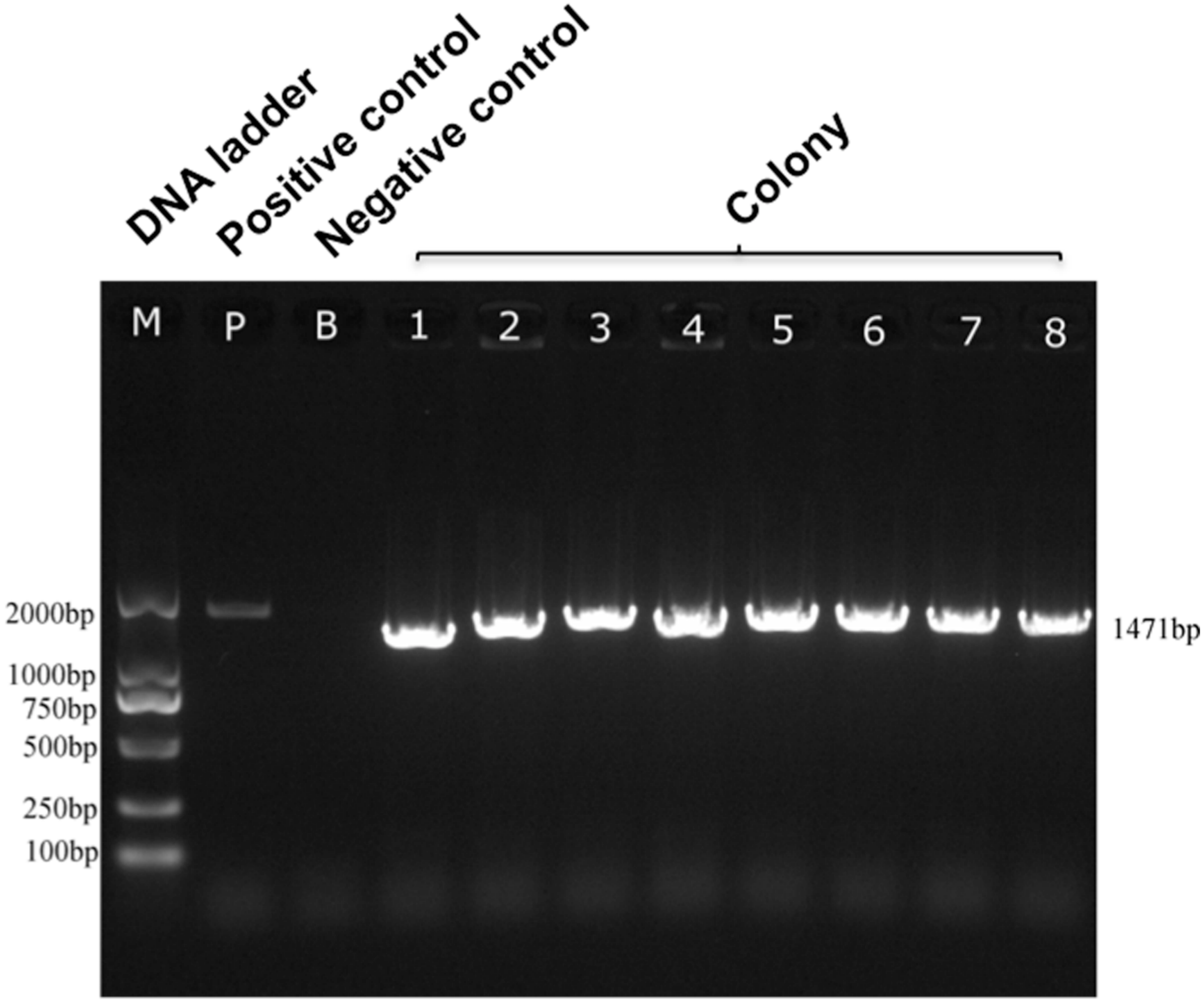

Supplementary Figure 1: Identification of positive bacteria integrated with porcine KAT8 cDNA by PCR assay. Detection of positive bacteria integrated with porcine *KAT8* cDNA. Single bacterial colony was picked up separately and their lysis solution was used to conduct common PCR assay. PCR products were analyzed by agarose gel electrophoresis to confirm the presence and size of p*KAT8* cDNA sequence. M: DNA marker D2000; P: positive control, namely cDNA of porcine blastocysts; B: negative control, namely water; 1-8: single bacterial colony.

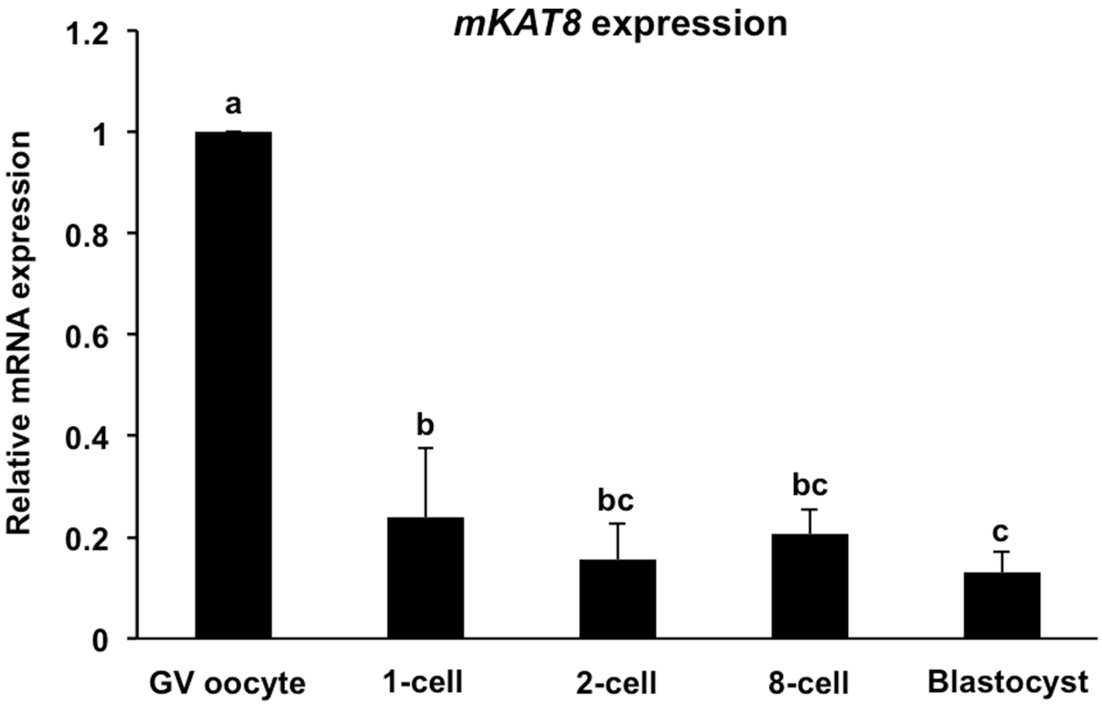

Supplementary Figure 2: Expression pattern of KAT8 mRNA in mouse oocytes and early embryos. The abundance of mouse *KAT8* transcripts from gene microarray data (6693234, GEO profiles in NCBI) was analyzed in GV oocytes and different stages of embryos including 1-cell, 2-cell, 8-cell and blastocysts. Data are shown as mean ± S.E.M and different letters across stages indicate significant differences ($P < 0.05$).

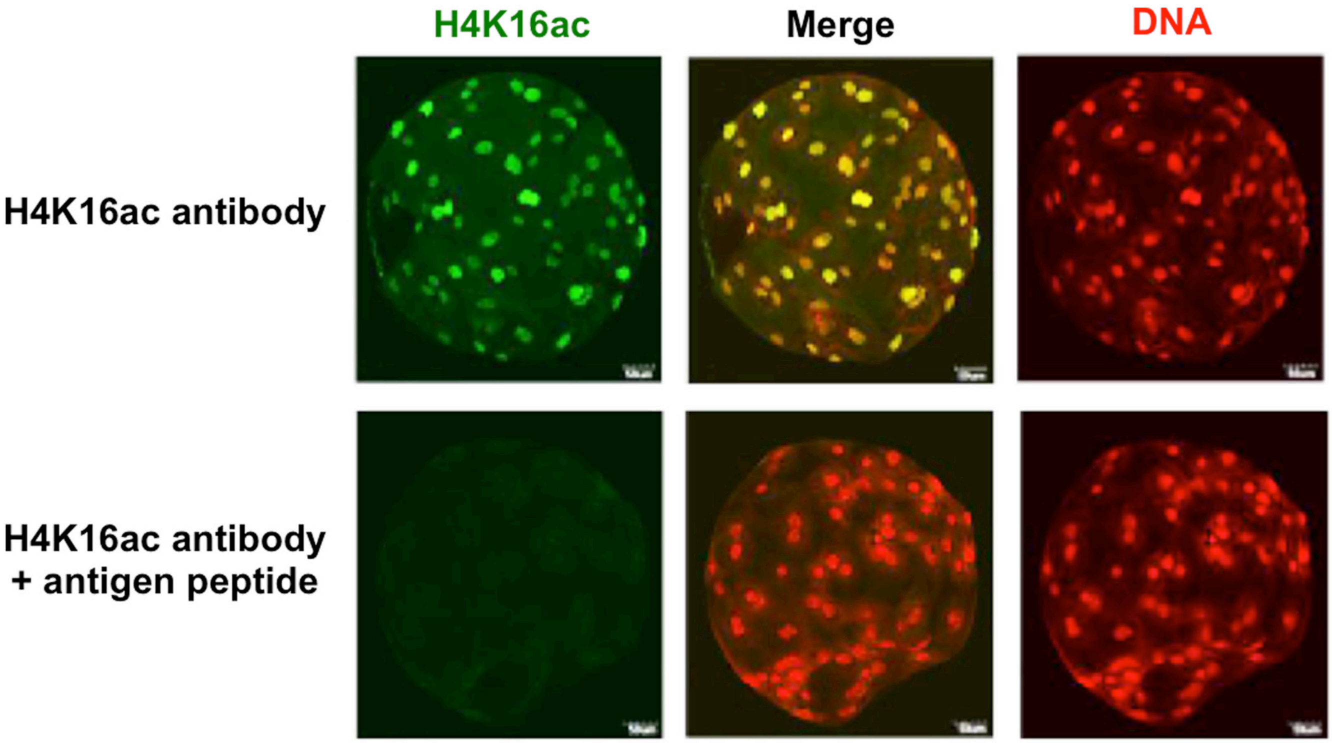

Supplementary Figure 3: Verification of H4K16ac antibody specificity. The commercially available H4K16ac primary antibody was preincubated without or with antigen peptide (Abcam, catalog no. ab154464, v/v=5:1) at room temperature for 1.5 h before the incubation with PA blastocysts. H4K16ac signal was observed in blastocysts using unabsorbed primary antibody. In contrast, H4K16ac signal was absent in blastocysts using pre-absorbed primary antibody. H4K16ac (green), DNA (red). Middle panel shows the merged images (yellow) between H4K16ac signal and DNA staining. Scale bars: 50 µm.

Supplementary Table 1: Homologous analysis of porcine, human and mouse KAT8

Length of AA	Identity against human (%)	Identity against mouse (%)
AA (458)	99%	98%

Supplementary Table 2: Porcine-specific primer sequences used in this study

Purpose	Gene	Primer sequences (5'-3')	PCR product	Gene ID
qPCR	p*KAT8*	F: AGATGACGAGTATCACCCAGAA<br>R: TGAGATGCTCCTCCACCAG	119 bp	NM-001284366
Full-length cloning	p*KAT8*	F: GTCACTTCCACTACAGCGATG<br>R: ATGACGAAACGGGTCTGT	1471bp	NM-001284366
Endogenous reference	*EF1α1*	F: AATGCGGTGGGATCGACAAA<br>R: CACGCTCACGTTCAGCCTTT	120 bp	NM_001097418-1

F: forward; R: reverse.

Supplementary Table 3: Sequence information for porcine *KAT8* siRNA and negative control siRNA

Gene	siRNA sequences (5'-3')	Start position
KAT8	F: GCAAAGACCACAAGAUUUATT	775bp
	R: UAAAUCUUGUGGUCUUUGCTT	
	F: CCACAAGACGCUCUACUUUTT	833bp
	R: AAAGUACAGCGUCUUGUGGTT	
Negative control	F: UUCUCCGAACGUGUCACGUTT	
	R: ACGUGACACGUUCGGAGAATT	

F: forward; R: reverse.
